# DNA barcoding and phylogeography of the *Hoplias malabaricus* species complex

**DOI:** 10.1038/s41598-022-09121-z

**Published:** 2022-03-28

**Authors:** Karen Larissa Auzier Guimarães, Marcos Prado Lima, Diego José Santana, Mendelsohn Fujiie Belém de Souza, Rômulo Sarmento Barbosa, Luís Reginaldo Ribeiro Rodrigues

**Affiliations:** 1 Programa de Pós-Graduação em Recursos Naturais da Amazônia (PPGRNA), Universidade Federal do Oeste do Pará (UFOPA), Santarém, Pará Brazil; 2 Programa de Pós-Graduação em Biodiversidade e Biotecnologia (REDE BIONORTE), Universidade Federal do Oeste do Pará (UFOPA), Santarém, Pará Brazil; 3 Laboratório de Genética e Biodiversidade (LGBio), Universidade Federal do Oeste do Pará (UFOPA), Santarém, Pará Brazil; 4 Laboratório de Biologia Molecular Animal, Instituto de Ciências e Tecnologia das Águas (ICTA), Universidade Federal do Oeste do Pará (UFOPA), Santarém, Pará Brazil; 5 Instituto de Biociências, Universidade Federal de Mato Grosso do Sul (UFMS), Campo Grande, Mato Grosso do Sul Brazil

**Keywords:** Biodiversity, Ecological genetics, Population genetics, Ichthyology

## Abstract

*Hoplias malabaricus* (Bloch, 1794) is a carnivorous fish species widely distributed from northern to southern South America. This taxon is believed to be a good model for the investigation of biogeographic events that shape the ichthyofauna evolution in the Neotropical freshwater systems. However, many studies have revealed that *H. malabaricus* hides a species complex that hampers its taxonomic identity and limit its practical value for evolutionary and biogeographic studies. In this paper, we used the mitochondrial gene *cytochrome c oxidase subunit I* (COI) to delimit cryptic species and explore the phylogeography of *H. malabaricus* sensu stricto. We found genetic evidence for putative new species in the genus *Hoplias* and showed that *H. malabaricus* (Bloch, 1794) is a major clade assigned to barcode index number (BIN) BOLD:ABZ3047. This species is structured in six subpopulations differentiated by high Fst values and restricts gene flow. The subpopulations of the São Francisco/East Atlantic/Eastern Northeast Atlantic/Parnaíba/Itapecuru River basins and Tapajós River Basin were the most differentiated and showed demographic fluctuations. The present distributional pattern is most likely explained through a scenario from the Pleistocene.

## Introduction

The trahiras are carnivorous fish of the family Erythrinidae (Characiformes) classified into three genera: *Erythrinus* Scopoli 1777, *Hoplerythrinus* Gill, 1896 and *Hoplias* Gill, 1903. The latter encompasses 15 species tentatively arranged in three species groups: the *Hoplias aimara* group, *H. lacerdae* group and *H. malabaricus* group. In this study we adopted the taxonomy of *H. malabaricus* group as Cardoso and colleagues^1^: *H. malabaricus* (Bloch, 1794), *H. microlepis* (Günther, 1864), *H. teres* (Valenciennes, 1847), *H. mbigua* Azpelicueta, Benítez, Aichino & Mendes, 2015, *H. misionera* Rosso, Mabragaña, González-Castro, Delpiani, Avigliano, Schenone & Díaz de Astarloa, 2016, and *H. argentinensis* Rosso, González-Castro, Bogan, Cardoso, Mabragaña, Delpiani & Díaz de Astarloa, 2018.

*Hoplias malabaricus* is widespread in the South American hydrographic basins and is frequently recorded along the Orinoco, Amazonas, São Francisco, Paraná, Paraguay and Prata river basins, where it inhabits lentic waters from lagoons, rivers and reservoirs^2–4^.

Cytogenetic data revealed karyotypic polymorphisms within *H. malabaricus* populations with chromosome morphology and diploid number variation (2n = 39–42), differentiated sex chromosome systems and other cytogenetic markers such as C-bands and Ag-NORs^2,5,6^. In the Amazon basin, five distinct karyomorphs (types A, C, E, F and G) were detected, including some highly dispersed through neighboring basins (types A, C and F) and others restricted (types E and G)^2^. Chromosomes were important to demonstrate intraspecific divergence of *H. malabaricus* populations and raise the hypothesis of a species complex hidden in this taxon^2,5–7^ that was corroborated with molecular data^8,9^.

Progress in molecular systematics has directly contributed to improving the species discovery and taxonomy resolution^10–12^. DNA barcoding is a high throughput method that uses a standardized sequence of the mitochondrial gene *cytochrome c oxidase subunit I* (COI) to delimit biological species and also to reveal if there is a phylogenetic structure within a species^13^, which has been successfully applied to address taxonomic questions of the Neotropical ichthyofauna^10,11,14^. DNA barcoding revealed markedly deep divergence of COI sequences between *H. malabaricus* populations, which supports the hypothesis that some mitochondrial lineages may constitute putative independent species^1,9^.

Cardoso and colleagues^1^ delimited 16 mitochondrial lineages of the *H. malabaricus* species complex to further investigate their taxonomic status. Recently, three of these lineages (BINs: ACO5223, AAZ3734, AAB1732) were recognized as valid species: *H. mbigua*, *H. misionera* and *H. argentinensis*^15–17^. On the other hand, the remaining cryptic diversity enclosed in the *H. malabaricus* complex wait for investigation. At least seven of the *H. malabaricus* mitochondrial lineages were recorded in the Amazon basin (BINs: AAB1732, ABZ3046, ABZ3047, AAB1731, ACF3787, ACK2158 and ADG3393).

Understanding population divergences on a regional scale is invaluable to disentangle intricate questions about the Neotropical ichthyofauna evolution. Widely distributed fish species, such as *H. malabaricus*, can be considered suitable models for biogeographic studies because they are exposed to extensive evolutionary and ecological drives that promote dispersal events^18–20^. On the other hand, the presence of cryptic species in the *H. malabaricus* complex is a confounding factor that limits its value for phylogeographic and population genetic studies. Additionally, a precise delimitation of cryptic species may strengthen the inferences in both basic and applied research fields, for example in conservation measures, ecological risk assessment and climate change effects on biodiversity^21^.

In this study, we assembled an extensive database of DNA barcodes including new sequences from the Amazon basin and adjacent drainages, aiming to delimit mitochondrial lineages representative of formally described species and discriminate putative cryptic species of the *H. malabaricus* complex. We delimited *H. malabaricus* (Bloch, 1794) “sensu stricto” following criteria in Cardoso and colleagues^1^ and evaluated it for genetic structure, phylogeography and demographic history.

## Methods

### Ethics statements

Fish specimens were collected under a Brazilian Government of the Sistema de Autorização e Informação em Biodiversidade (SISBIO), permission N. 24384-1. For tissue and voucher preservation, the specimens were anesthetized and euthanized by exposure in eugenol solution for a few minutes until the complete stop of the opercula beats. All procedures were approved by Comissão de Ética no Uso de Animais (CEUA) of Universidade Federal do Oeste do Pará (N 09003/2016) and followed all relevant guidelines. Additionally, the study was carried out in compliance with ARRIVE guidelines.

### Sampling and study area

We sampled 153 *H. malabaricus* specimens from 38 localities in the Amazon Basin (Brazil, Peru), the Araguaia-Tocantins basin (Brazil), the Western Northeastern Atlantic Basin (Brazil), the Guiana Shield drainages (Guiana) and the Orinoco Basin (Venezuela, Colombia). The fish were collected using seine nets, casting nets and fish hooks. Samples of epaxial muscle were preserved in absolute ethanol and stored at -20 °C. The specimens were fixed with 10% formalin for 48 h, washed and preserved in 70% ethanol. Specimens were later deposited in the Fish Collection of the Instituto de Ciências e Tecnologia das Águas and the Laboratório de Genética e Biodiversidade, Universidade Federal do Oeste do Pará (Brazil).

### DNA extraction, PCR and sequencing

DNA extraction followed a salting-out protocol and the amounts were evaluated in a 1% agarose gel stained with Gelred (Biotium)^22,23^. DNA fragments of *cytochrome c oxidase subunit I* (COI) mitochondrial gene were amplified using the standard DNA barcoding primers Fish F1 and Fish R1^24^. The reactions were assembled in 25 μL, containing 15 μL sterile H_2_O, 2.8 μL dNTP mix (1.25 mM), 2.5 μL buffer 10 × (200 mM Tris–HCl (pH 8.4) + 500 mM KCl), 2.5 μL MgCl_2_ (50 mM), 0.5 μL of each primer (5 μM), 0.2 μL Taq DNA polymerase (5 U/μL) and 1 μL of genomic DNA (50–100 ng). The cycling profile was as follows: 95 °C/2 min, 35 cycles of 94 °C/30 s, 54 °C/30 s and 72 °C/1 min, and a final step of 72 °C/10 min. The amplifications were performed with a Pxe 0.2 thermocycler (Thermo Scientific) and the amplified products were evaluated in a 1% agarose gel stained with Gelred. PCR products were purified with PEG8000 protocol^25^. COI sequences were obtained by the Sanger method using the ABI PRISM Big Dye Terminator V.3 Cycle Sequencing kit (Applied Biosystems, Waltham, Massachusetts, USA). Sequencing reactions were perfomed in 96-well plates with a final volume of 10 μL, containing 5 μL of sterile H_2_O, 1.5 μL of sequencing buffer 5 ×, 0.5 μL of primer (10 μM), 1 μL of Big Dye mixture and 2 μL of cleaned PCR. The dye incorporation reactions followed 96 °C/1 min; 35 cycles of 96 °C/15 s, 50 °C/15 s, and 60 °C/4 min. The plates were precipitated in ethanol/EDTA, eluted with 10 μL Formamida Hi-Di and detected with an ABI 3500 genetic analyser (Applied Biosystems), following the manufacturer’s instructions.

### Molecular data analysis and species delimitation

The DNA barcode dataset was enriched with 286 COI sequences downloaded from the public repositories: Barcode of Life Database (https://www.boldsystems.org) and GenBank (https://www.ncbi.nlm.nih.gov). To obtain higher accuracy for cryptic species delimitation with barcodes we included sequences representative of *H. malabaricus*, *H. misionera*, *H. mbigua*, *H. microlepis*, *H. argentinensis*, *H. intermedius*, *H. aimara*, *H. australis*, *H. lacerdae* and *H. curupira*. The geographic coverage of the *H. malabaricus* complex was enlarged in the following regions: Lower Amazonas Rivers^9,11^, the East Atlantic, the Eastern Northeast Atlantic, Parnaíba River, São Francisco and the Itapecuru River basins^8^ the Madeira River in Brazil and Bolivia, and drainages from the Guiana shields in Surinam and French Guiana^1^. Detailed information on the DNA barcoding dataset and specimen metadata is listed in Supplementary Data S1.

The consensus sequences were assembled using Geneious R7 (Biomatters, New Zealand) and aligned with Clustal W v1.4^26^. We used GBLOCKS v0.91b^27^ to inspect the alignment and trim the sequence tips and poorly aligned regions. The sequences generated in this work were deposited in a DNA barcoding repository (http://www.boldsystems.org) linked to the Project “Amazonian Trahiras (AMTRA)” (see Supplementary Data S2).

We delimited species by: (1) barcode index number (BIN)^28^, (2) generalized mixed Yule coalescent (GMYC)^29,30^ and (3) automatic barcode gap discovery (ABGD)^31^. The BIN analysis is an automated process implemented in the platform www.boldsystems.org, which is based on genetic distances to identify clusters of query sequences against a DNA barcode library; such clusters are coded as BIN numbers and interpreted as operational taxonomic unit (OTUs) that represent a species. To perform the GMYC analysis, we removed haplotype duplicates with ElimDupes (https://www.hiv.lanl.gov/content/sequence/elimdupesv2/elimdupes.html) and made an ultrametric tree using BEAST v1.8.0^32^, following these parameters: evolutionary model HKY + I + G chosen with jModelTest^33^, molecular clock lognormal relaxed, Yule speciation process. *Leporinus amblyrhynchus* Garavello & Britski 1987 was adopted as the outgroup. We ran a Bayesian reconstruction with 80 million MCMC iterations, sampled every 1000 iterations with a burn-in of 10%. The tree convergence and stability were checked with the software Tracer v.1.7.1^32^, retaining an effective sample size (ESS) > 200. The trees were combined with TreeAnotator v1.8.0^32^, and the output file was saved in Newick tree format to be used for GMYC delimitation. The analysis of coalescence/speciation (GMYC) was processed following the model single threshold, in the environment R 3.4.3^34^ supplemented with libraries *Splits* (Species Limits by Threshold Statistics)^35^ and *Ape* (Analyses of Phylogenetics and Evolution in R language)^36^. ABGD was processed on the platform www.bioinfo.mnhn.fr/abi/public/abgd/abgdweb.html using the alignment data set (fasta file) as the input file. We set the parameters: model K80, Pmin. 0.001, Pmax 0.01 and barcoding gap width X = 0.2.

To integrate the phylogenetic information, species delimitation and divergence time we processed a second Bayesian reconstruction following the procedures mentioned above with minor modifications: 200 million MCMC iterations sorted at each 1000 and 10% of burn-in and a strict clock model. Divergence times were calibrated with a mutation rate of 1% per million years (Myr), which is conservative for fish mtDNA^37,38^. The resulting trees were assembled with TreeAnotator and the topology was visualized/edited with FigTree v1.2.2 (http://tree.bio.ed.ac.uk/software/figtree/). Pairwise genetic distances between delimited species were measured following the K80 model^39^ using the software MEGA X software^40^.

### Population genetics and phylogeography

The individuals assumed to be *Hoplias malabaricus* sensu stricto (BIN ABZ3047) following designation proposed in Cardoso and colleagues^1^ were investigated for intraspecific genetic diversity and population structure. We used the software GENELAND R package v. ≥ 4.0.0 (http://www2.imm.dtu.dk/gigu/Geneland/)^41^ implemented with *R* v3.4.0^34^ to investigate population subdivisions and to find the geographic population units, based on Bayesian statistics. The population genetic structure was evaluated through F_ST_ statistics and molecular variance analysis (AMOVA) implemented with Arlequin v.3.1^42^. We assumed populations as the clusters of individuals such as revealed by Geneland analysis. For F_ST_ divergence, we follow Wright and colleagues^43^ categories: low (0.00–0.05), moderate (0.05–0.15), high (0.15–0.25) and elevated (> 0.25). Parameters of the population genetics (e.g. haplotypes, nucleotide diversity, polymorphic sites) were analysed with DNAsp v.6^44^. A haplotype network was constructed based on median joining algorithm^45^ with the assistance of PopART software^46^.

To explore the demographic history, we applied neutrality tests Tajima’s D^47^ and Fu’s Fs^48^, implemented with Arlequin v.3.1^42^. Additionally, to detect population size variations we investigated the mismatch distributions and Bayesian skyline plot (BSP). These analyses were implemented with DNAsp v.6^44^ and BEAST v.1.8.0^32^. BSP analysis adopted the HKY + I + G model and 100 million MCMC sorted each 1000 iterations.

We constructed an ecological niche model with the maximum entropy algorithm MAXENT version 3.3.3 k^49–51^ based on 82 georeferenced occurrence points (Fig. 6a) and 19 bioclimatic variables from WorldClim (https://www.worldclim.org/data/bioclim.html). Such variables were correlated with a 2.5 arc-minute spatial scale^51^, and the distributional limits were assessed from the median occurrence with 50 bootstrap pseudoreplicates. The theoretical distributional patterns were visualized with QGIS v.3.16.8-Hannover (Quantum GIS Development Team, www.qgis.org). We used jackknife permutations to evaluate the model performance gain and to identify and retain the most relevant explanatory variables.

## Results

### DNA barcoding and species delimitation

We analysed 439 COI sequences from 10 species of the genus *Hoplias*. The sequences were 621 bp long without stop codons or indels. The dataset showed a base composition of 29.4% (T), 29.5% (C), 23.3% (A) and 17.7% (G).

**Figure 1:**
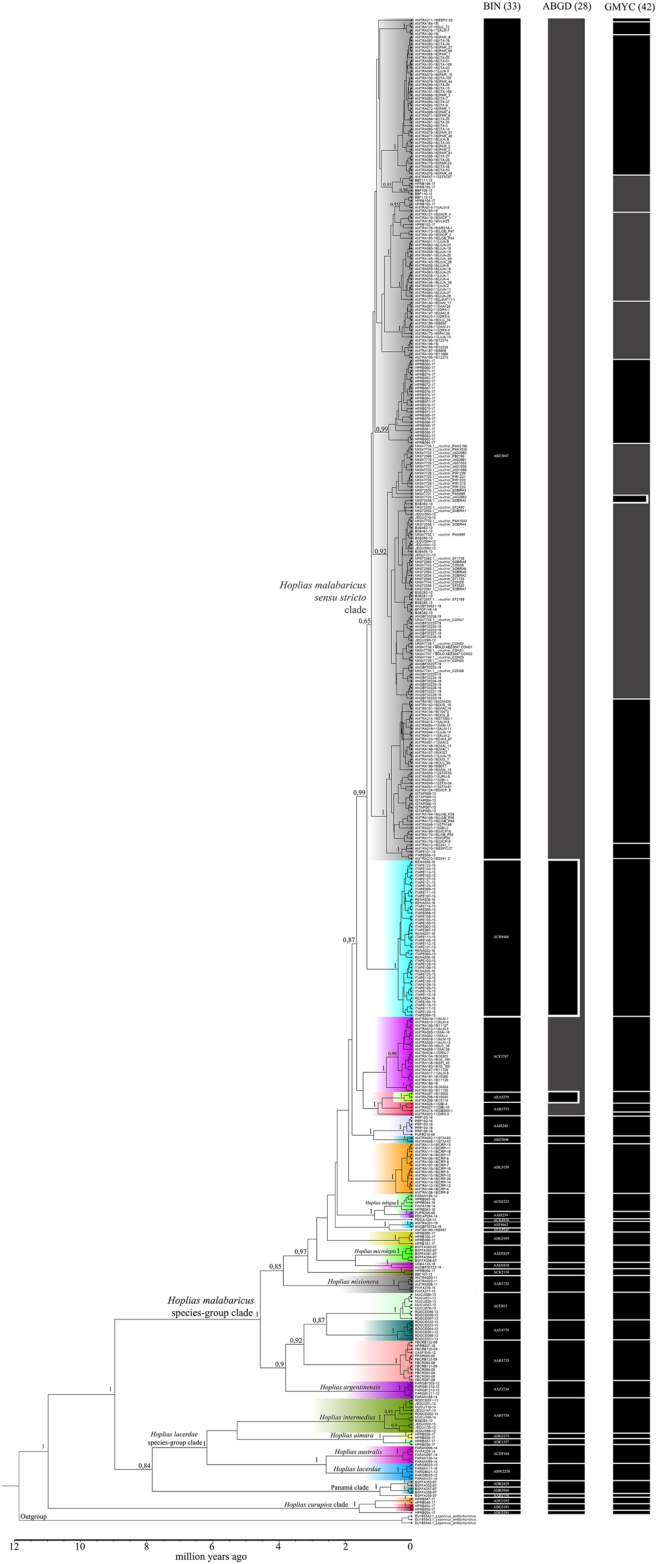
COI mitochondrial gene tree recovered from a Bayesian inference analysis in the genus *Hoplias* (Erythrinidae). All clades highlighted are the evolutionary lineages recovered in BIN species delimitation. Black bars on the right show the partitions inferred as putative species delimited through, BIN, ABGD and GMYC analysis. Values in the nodes indicate statistical support of posterior probability. The tree was drawn with FigTree v1.2.2 (http://tree.bio.ed.ac.uk/software/figtree/).

Phylogenetic Bayesian inference showed three major clades in *Hoplias*. The largest clade encompasses the species assembled to the *H. malabaricus* group that emerged more recently; the second clade is configured with the species from the *H. lacerdae* group, which surprisingly nested five individuals deposited as *H. malabaricus* (Panamá clade); the most basal clade is represented by *Hoplias curupira* lineages (Fig. 1). All the lineages received strong support from the Bayesian genealogic inference (100% posterior probability—PP), with the exception of the *H. malabaricus* lineages that were assembled to BIN ABZ3047 supported by 65% PP (Fig. 1).

Based on the present DNA barcode library of *Hoplias* we delimited a varied number of species with different methods: ABGD (28sp), BIN (33sp) and GMYC (42 sp). The pairwise genetic distances between groups (BINs) ranged from 1.1 to 21.5%, while the intra-BIN distances ranged from 0 to 1.6% (Table 1). The largest distances were detected for *Hoplias curupira* lineages. We found total congruence for delimiting six groups that match unequivocally to species: *H. lacerdae*, *H. australis*, *H. intermedius*, *H. argentinensis*, *H. misionera* and *H. microlepis*. Additionally, 17 putative new species were delimited with all the methods applied (BIN, ABGD and GMYC). On the other hand, discrepant species delimitation was observed and showed taxonomic uncertainty on the following species or clades: *H. malabaricus* (Panama Clade) was delimited with three species (BIN ADR2428, ADR3966 and AAB1730) but with two species (ABGD, GMYC). *H. mbigua* was delimited with three species (BIN, GMYC) (ACO5223, AAI8239 and ACK8866) but with two species (ABGD). The specimens assigned to *H. malabaricus* were assembled in 11 putative species (BINs: ADG3393, AEA4944, AEF4663, ADL3159, ABZ3046, AAI8240, AAB1731, AEA5279, ACF3787, ACR9466 and ABZ3047); however, ABGD supported eight species while GMYC delimited 21 species hidden in this taxon. Therefore, the *H. malabaricus* species complex was clearly evidenced from distinct DNA barcode analysis.

**Table 1:** Pairwise genetic distances (K2P) between and within species of *Hoplias* based on COI sequences.

Groups barcode index number (species) [n]		1	2	3	4	5	6	7	8	9	10	11	12	13	14	15	16	17	18	19	20	21	22	23	24	25	26	27	28	29	30	31	32	33	34
1	ABZ3047 [247]	**0.016**																																	
2	AAB1733 [12]	0.080	**0.002**																																
3	AAD3630 [2]	0.044	0.074	**0.013**																															
4	AAY4779 [6]	0.087	0.066	0.078	**0.004**																														
5	ACI3811 [8]	0.094	0.084	0.086	0.065	**0.003**																													
6	ACR9466 [46]	0.028	0.082	0.051	0.088	0.091	**0.001**																												
7	AEF4663 [2]	0.043	0.077	0.054	0.087	0.099	0.053	**0**																											
8	AAB1731 [4]	0.025	0.071	0.048	0.076	0.092	0.032	0.043	**0.009**																										
9	ABZ3046 [2]	0.034	0.080	0.042	0.079	0.100	0.039	0.041	0.030	**0.002**																									
10	ACF3787 [22]	0.026	0.075	0.042	0.075	0.085	0.035	0.041	0.019	0.024	**0.004**																								
11	ADL3159 [15]	0.030	0.075	0.043	0.082	0.088	0.031	0.032	0.030	0.029	0.028	**0.001**																							
12	AEA9444 [1]	0.039	0.076	0.051	0.085	0.094	0.047	0.015	0.038	0.035	0.036	0.030	−																						
13	ADG3393 [4]	0.063	0.088	0.067	0.087	0.096	0.066	0.057	0.062	0.064	0.059	0.057	0.056	**0.004**																					
14	ACK2158 [2]	0.061	0.089	0.061	0.092	0.100	0.061	0.052	0.058	0.045	0.053	0.046	0.058	0.074	**0.005**																				
15	ACK8876 [1]	0.058	0.098	0.064	0.096	0.097	0.070	0.055	0.055	0.051	0.050	0.049	0.050	0.079	0.075	−																			
16	AAI8240 [6]	0.040	0.075	0.054	0.079	0.108	0.047	0.051	0.031	0.026	0.036	0.040	0.047	0.072	0.055	0.063	**0.001**																		
17	AAI8239 [2]	0.044	0.071	0.044	0.074	0.072	0.054	0.041	0.041	0.037	0.036	0.037	0.036	0.062	0.059	0.030	0.045	**0**																	
18	AEA5279 [3]	0.032	0.083	0.052	0.087	0.103	0.039	0.046	0.022	0.036	0.030	0.036	0.047	0.064	0.062	0.057	0.043	0.045	**0.003**																
19	AAZ3734 (*H. argentinensis*) [5]	0.072	0.075	0.070	0.080	0.081	0.074	0.070	0.073	0.075	0.069	0.074	0.072	0.091	0.073	0.094	0.076	0.075	0.077	**0.001**															
20	ACO5223 (*H. mbigua*) [5]	0.044	0.078	0.046	0.079	0.076	0.052	0.038	0.043	0.035	0.038	0.032	0.034	0.063	0.057	0.031	0.050	0.011	0.046	0.075	**0.003**														
21	AAD3629 (*H. microlepis*) [5]	0.055	0.081	0.038	0.082	0.080	0.060	0.060	0.055	0.049	0.050	0.057	0.059	0.077	0.057	0.071	0.061	0.050	0.060	0.066	0.052	**0.003**													
22	AAB1732 (*H. misionera*) [5]	0.071	0.081	0.064	0.078	0.089	0.076	0.060	0.076	0.071	0.070	0.068	0.063	0.077	0.082	0.086	0.081	0.065	0.079	0.074	0.064	0.082	**0.004**												
23	ADR3966 [2]	0.171	0.157	0.158	0.152	0.160	0.169	0.159	0.165	0.167	0.160	0.170	0.171	0.168	0.152	0.193	0.175	0.167	0.157	0.147	0.168	0.155	0.155	**0.002**											
24	AAB1730 [1]	0.169	0.154	0.155	0.151	0.157	0.165	0.156	0.161	0.168	0.158	0.166	0.167	0.173	0.148	0.189	0.172	0.164	0.153	0.148	0.166	0.156	0.154	0.024	–										
25	ADR2428 [2]	0.168	0.153	0.155	0.147	0.150	0.167	0.157	0.160	0.162	0.155	0.168	0.168	0.172	0.147	0.184	0.171	0.159	0.157	0.147	0.159	0.155	0.148	0.015	0.021	**0.002**									
26	AAB1734 (*H. intermedius*) [10]	0.143	0.162	0.149	0.147	0.138	0.146	0.156	0.144	0.154	0.140	0.150	0.160	0.148	0.162	0.176	0.162	0.154	0.146	0.137	0.152	0.162	0.131	0.158	0.166	0.158	**0.009**								
27	ADG3392 (*H. curupira*) [2]	0.180	0.200	0.188	0.198	0.199	0.173	0.189	0.183	0.186	0.176	0.171	0.193	0.181	0.190	0.204	0.181	0.186	0.185	0.185	0.183	0.184	0.197	0.209	0.215	0.216	0.184	**0**							
28	ADG3181 (*H. curupira*) [2]	0.182	0.198	0.186	0.199	0.195	0.173	0.175	0.187	0.187	0.178	0.175	0.184	0.192	0.195	0.199	0.184	0.182	0.190	0.181	0.177	0.180	0.195	0.214	0.215	0.212	0.195	0.035	**0**						
29	ADG3391 (*H. curupira*) [1]	0.153	0.181	0.163	0.175	0.186	0.150	0.165	0.156	0.158	0.152	0.143	0.164	0.161	0.162	0.182	0.155	0.161	0.156	0.173	0.159	0.161	0.185	0.205	0.203	0.209	0.183	0.021	0.040	–					
30	ACD9164 (*H. australis*) [5]	0.153	0.162	0.161	0.149	0.152	0.165	0.151	0.159	0.163	0.156	0.166	0.159	0.148	0.161	0.182	0.166	0.158	0.162	0.140	0.156	0.153	0.151	0.147	0.143	0.143	0.112	0.176	0.184	0.179	**0.003**				
31	ABW2258 (*H. lacerdae*) [5]	0.147	0.147	0.153	0.136	0.134	0.156	0.142	0.150	0.161	0.148	0.155	0.153	0.129	0.165	0.175	0.160	0.154	0.157	0.140	0.153	0.158	0.135	0.146	0.145	0.146	0.113	0.170	0.179	0.170	0.059	**0.003**			
32	ADE1357 (*H. aimara*) [2]	0.168	0.167	0.167	0.171	0.169	0.179	0.166	0.170	0.160	0.161	0.173	0.172	0.176	0.177	0.192	0.164	0.162	0.172	0.163	0.165	0.171	0.154	0.164	0.171	0.176	0.119	0.204	0.221	0.201	0.149	0.138	**0**		
33	ADG3375 (*H. aimara*) [2]	0.159	0.168	0.159	0.170	0.159	0.173	0.156	0.160	0.159	0.151	0.164	0.157	0.175	0.177	0.178	0.159	0.149	0.162	0.162	0.151	0.165	0.145	0.172	0.170	0.175	0.126	0.199	0.206	0.190	0.150	0.136	0.022	**0.002**	
34	Outgroup [3]	0.214	0.240	0.196	0.209	0.218	0.211	0.198	0.216	0.215	0.214	0.211	0.214	0.223	0.193	0.227	0.216	0.205	0.214	0.203	0.209	0.210	0.201	0.228	0.226	0.229	0.210	0.224	0.231	0.217	0.213	0.212	0.234	0.223	**0.004**

Bold values indicate the intra-BIN distance. Number of individuals are shown in brackets.

**Figure 2:**
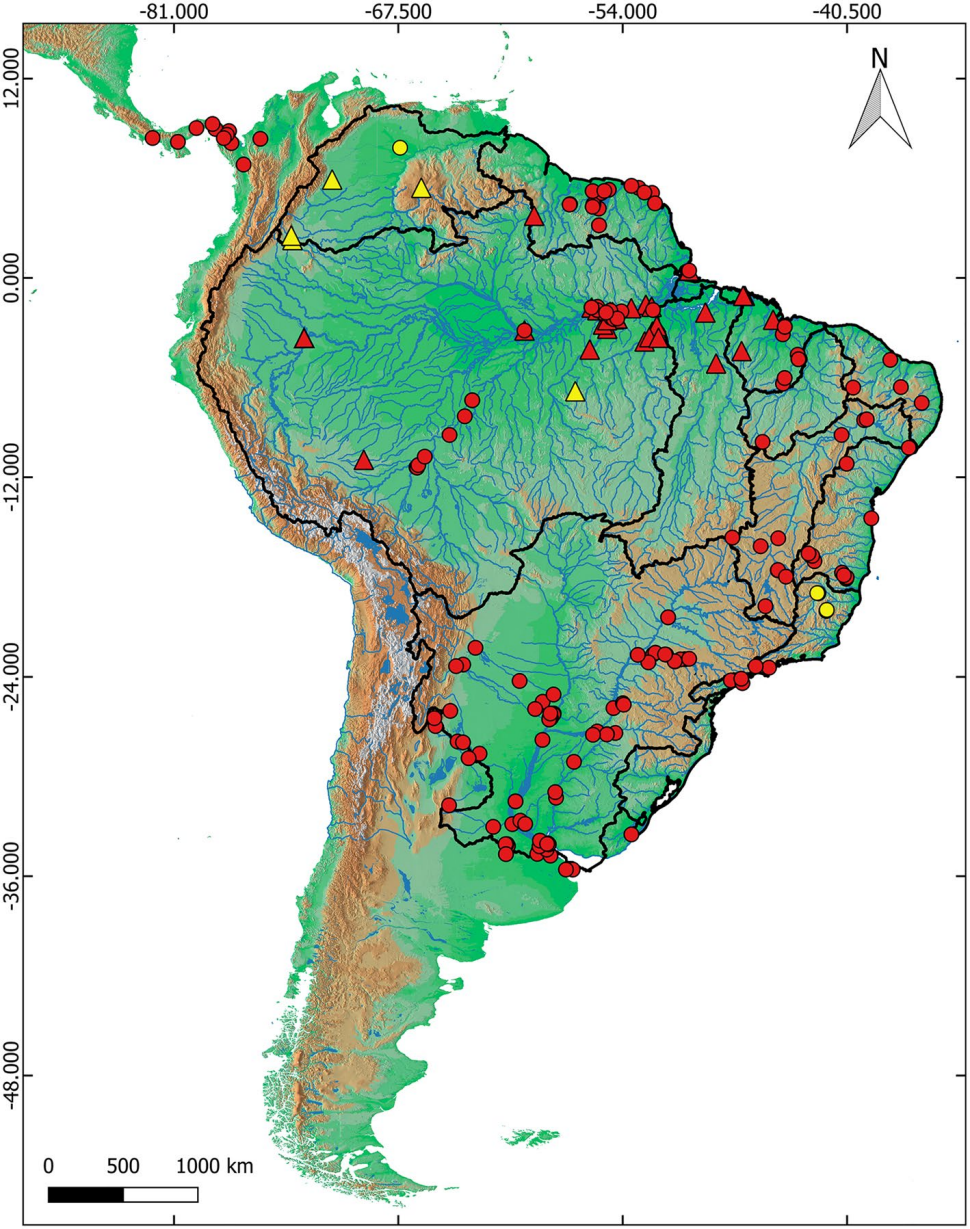
Geographic distribution of all genetic samples from species belonging to the *Hoplias malabaricus* species-group in South America. Localities sampled in this study (triangles) and localities of sequences mined from the internet (circles). The distribution of new putative species found in this study is in yellow. The map was done with QGIS v.3.16.8-Hannover (Quantum GIS Development Team, http://www.qgis.org).

We recognized *H. malabaricus* (Bloch, 1794) as the clade that nested the individuals from Suriname, since it is the type locality of this species. Such clade was delimited as BIN ABZ3047. To explore the intraspecific genetic relationships, we assumed BIN ABZ3047 as a single species, which is hereinafter referred to as *H. malabaricus *sensu stricto following Cardoso et al. (2018). This species comprises eleven entities widely distributed throughout the Amazon Basin and adjacent drainages, including a subclade that is restricted to the Guyana shield drainages. An updated distribution map of the *Hoplias malabaricus* species group based on records of barcoded individuals is provided and the geographic occurrence of news records of putative species is highlighted (Fig. 2).

### Population genetics and phylogeography of *H. malabaricus* sensu stricto

**Figure 3:**
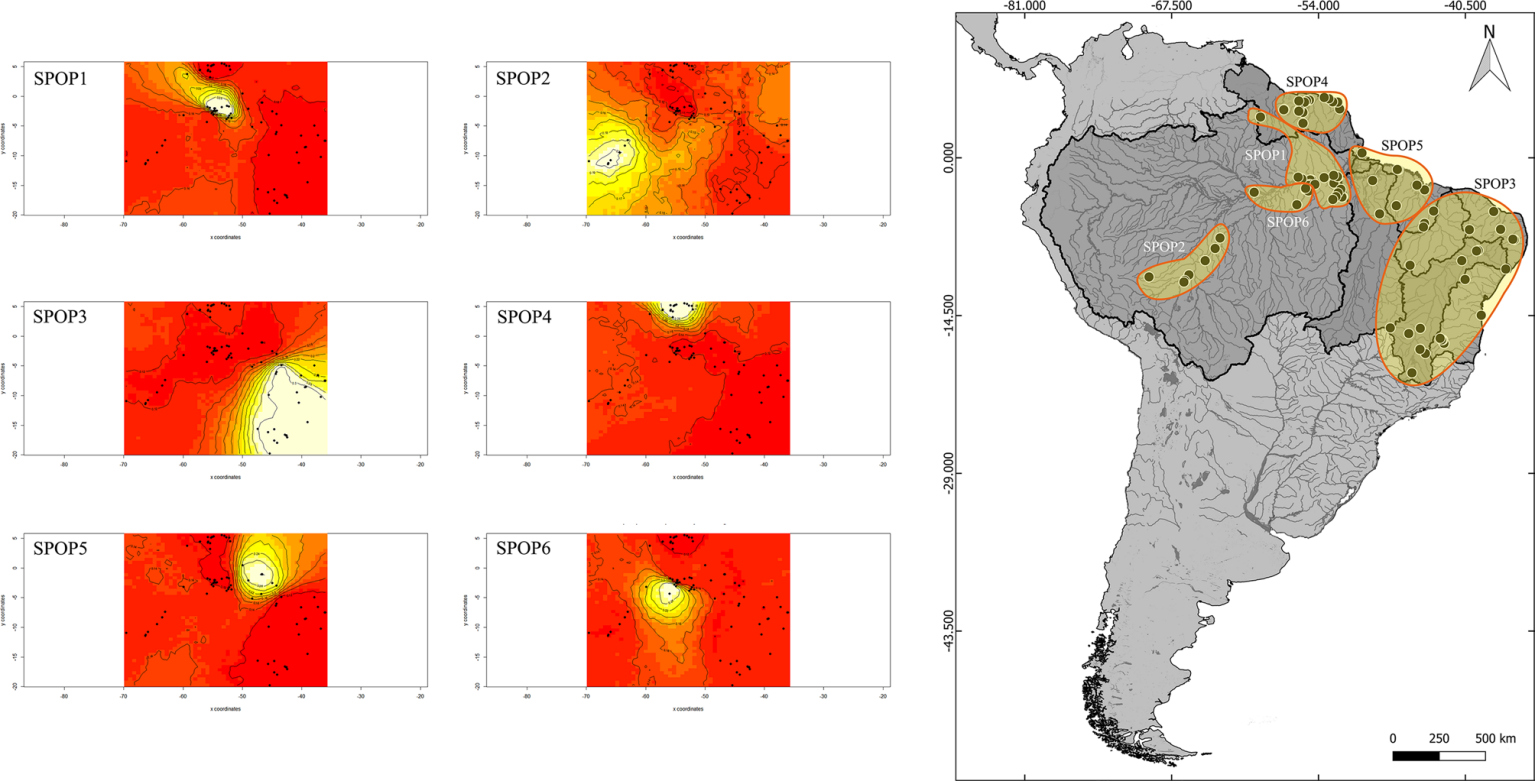
Subpopulations of *Hoplias malabaricus* (BIN ABZ3047) from the Amazon Basin and adjacent drainages inferred with COI haplotypes and geographic data using GENELAND R package v. ≥ 4.0.0 (http://www2.imm.dtu.dk/singrainageseam). The polygons shaded white indicate the largest probabilities of associating haplotypes (black dots) with populations. The map was done with QGIS v.3.16.8-Hannover (Quantum GIS Development Team, www.qgis.org).

Based on the DNA barcodes of 247 individuals we observed 79 polymorphic sites and 66 haplotypes (see Supplementary Data S3). The spatial variation of genetic data indicated a distribution of six subpopulations (SPOP): (1) Lower Amazonas River confluences/Xingu River/Rupununi River (SPOP1), (2) Madeira/Purus Rivers (SPOP2), (3) São Francisco/East Atlantic/Eastern Northeast Atlantic/Parnaíba/Itapecuru River basins (SPOP3), (4) Guiana Shields drainages (SPOP4), 5) Western-Northeast Atlantic/Tocantins basin (SPOP5), (6) Tapajós River Basin (SPOP6) (Fig. 3).

The *H. malabaricus* sensu stricto subpopulations presented high genetic diversity counting from 6 to 19 haplotypes and haplotypic diversity (h) from 0.230 (SPOP6) to 0.956 (SPOP2). The nucleotide diversity (π) was higher in SPOP1 (0.0187) and lowest in SPOP6 (0.0005). Two subpopulations (SPOP3 and SPOP6) resulted in negative and statistically significant Fu’s Fs and Tajima’s D values that were interpreted as evidence of neutrality deviation by purifying selection or population expansion. For the other subpopulations, both the neutrality tests suggested the long-term population stability (Table 2).

**Table 2:** Genetic diversity and values of neutrality tests of *H. malabaricus* subpopulations from the Amazon basin and adjacent drainages, based on mtDNA (COI gene).

Subpopulation	N	Ha	S	*H*	π	Fu's FS	p value	Tajima's D	p value
SPOP1	75	19	39	0.874	0.0187	− 0.09562	0.52300	− 0.21355	0.50100
SPOP2	10	8	16	0.956	0.0114	− 1.69595	0.15100	− 0.06384	0.48100
SPOP3	75	15	20	0.877	0.0047	− **6.44668**	**0.00800**	− **1.69915**	**0.01600**
SPOP4	25	9	15	0.760	0.0051	− 2.14428	0.10400	− **1.50352**	**0.04300**
SPOP5	20	11	21	0.947	0.0156	− 0.99411	0.35500	0.13010	0.59400
SPOP6	42	6	4	0.230	0.0005	− **4.38750**	**0.00000**	− **1.76047**	**0.01000**

**N** = individuals, **Ha** = haplotypes, **S** = polymorphic sites, **h** = haplotypic diversity, **π** = nucleotide diversity. Bold values are statistically significant (p < 0.05). Subpopulations: (1) Lower Amazonas River confluences/Xingu River/Rupuruni River (SPOP1), (2) Madeira/Purus Rivers (SPOP2), (3) São Francisco/East Atlantic/Eastern Northeast Atlantic/Parnaíba/Itapecuru River basins (SPOP3), (4) Guiana Shields drainages (SPOP4), (5) Western-Northeast Atlantic/Tocantins basin (SPOP5), (6) Tapajós River Basin (SPOP6).

**Figure 4:**
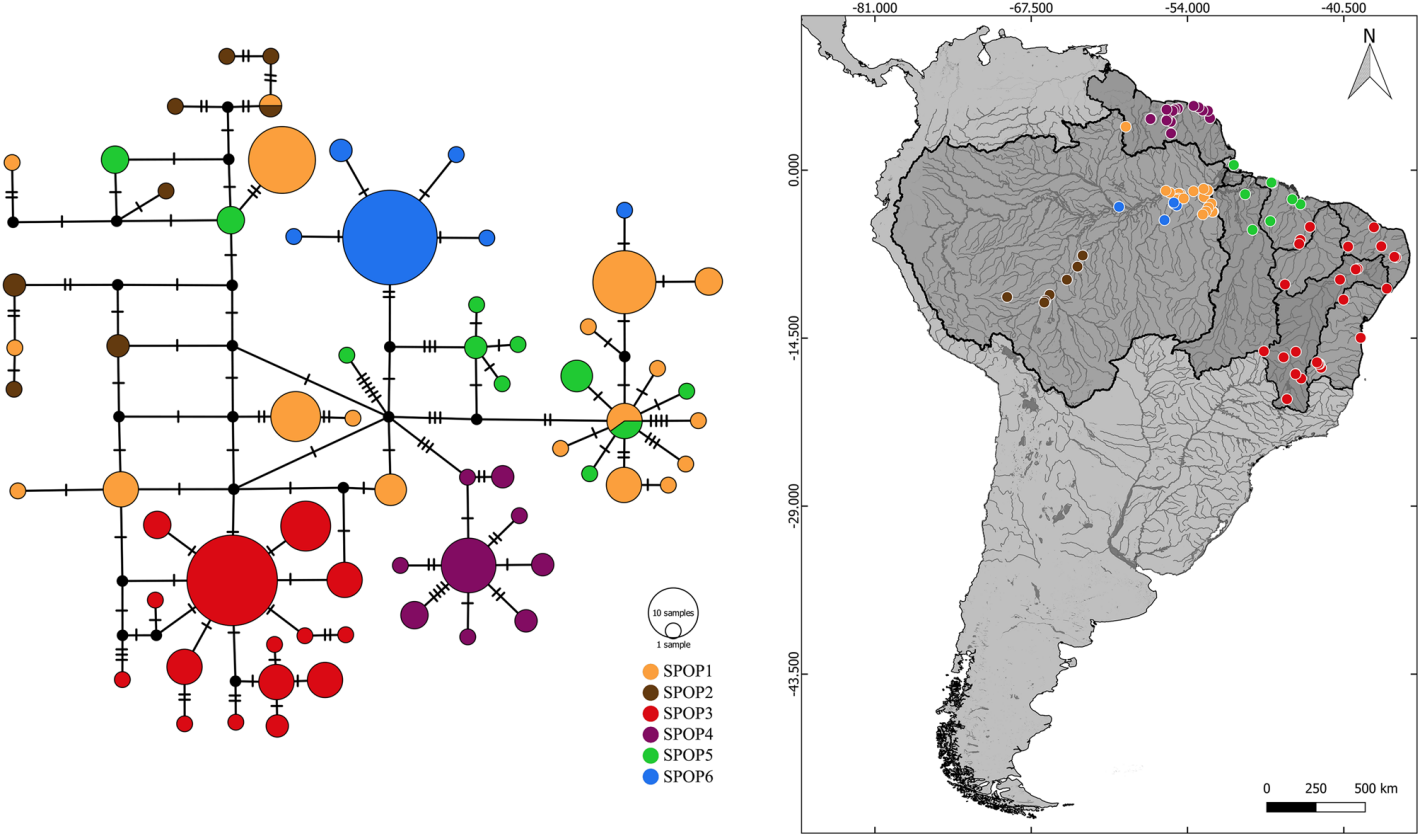
Median joining haplotype network of *Hoplias malabaricus* (BIN ABZ3047) based on the mitochondrial gene COI. The size of circles is proportional to haplotype frequency. Black circles indicate not sampled or possibly extinct haplotypes. Perpendicular bars show the mutational steps. Subpopulations: (1) Lower Amazonas River confluences/Xingu River/Rupuruni River (SPOP1), (2) Madeira/Purus Rivers (SPOP2), (3) São Francisco/East Atlantic/Eastern Northeast Atlantic/Parnaíba/Itapecuru River basins (SPOP3), (4) Guiana Shields drainages (SPOP4), 5) Western-Northeast Atlantic/Tocantins basin (SPOP5), (6) Tapajós River Basin (SPOP6). The network was drawn with PopART (http://popart.otago.ac.nz) and the map was done with QGIS v.3.16.8-Hannover (Quantum GIS Development Team, http://www.qgis.org).

The haplotype network showed that only two haplotypes were shared between *H. malabaricus* sensu stricto subpopulations (SPOP1-SPOP2 and SPOP1-SPOP5). Three haplogroups were depicted and coincide with SPOP3, SPOP4 and SPOP6, which showed only private haplotypes demonstrating higher genetic differentiation (Fig. 4).

A pairwise Fst comparison demonstrates that subpopulation SPOP6 is the most differentiated (Table 3). The lowest Fst value (0.407) was recorded for the SPOP6xSPOP1 comparison and the highest differentiation was detected to SPOP6xSPOP2 (0.545). AMOVA results demonstrated 27.20% of variation was detected among populations and 72.80% within populations (Table 4).

**Table 3:** Pairwise *F*_*ST*_ values between subpopulations of *Hoplias malabaricus stricto *sensu (BOLD:ABZ3047) based on mtDNA (COI gene) haplotypes.

Hierarchical level	*F*_*ST*_ matrix				
	SPOP1	SPOP2	SPOP3	SPOP4	SPOP5
SPOP1					
SPOP2	0.09022 (0.00391)				
SPOP3	0.17423 (0.00000)	0.15050 (0.00000)			
SPOP4	0.17667 (0.00000)	0.15419 (0.00000)	0.22995 (0.00000)		
SPOP5	0.09871 (0.00000)	0.06009 (0.01270)	0.15725 (0.00000)	0.15910 (0.00000)	
SPOP6	0.40667 (0.00000)	0.54463 (0.00000)	0.45698 (0.00000)	0.54259 (0.00000)	0.48650 (0.00000)

The numbers in brackets are p-values at a significance level of 0.05. Subpopulations: (1) Lower Amazonas River confluences/Xingu River/Rupuruni River (SPOP1), (2) Madeira/Purus Rivers (SPOP2), (3) São Francisco/East Atlantic/Eastern Northeast Atlantic/Parnaíba/Itapecuru River basins (SPOP3), (4) Guiana Shields drainages (SPOP4), (5) Western-Northeast Atlantic/Tocantins basin (SPOP5), (6) Tapajós River Basin (SPOP6).

**Table 4:** AMOVA results of *Hoplias malabaricus stricto *sensu from the Amazon basin and adjacent drainages.

Source of variation	d.f.	Sum of squares	Variance components	Percentage of variation	p value
Among populations	5	27.681	0.13687 Va	27.20	0.000
Within populations	240	87.936	0.36640 Vb	72.80	0.000
Total	245	115.618	0.50327		

**Figure 5:**
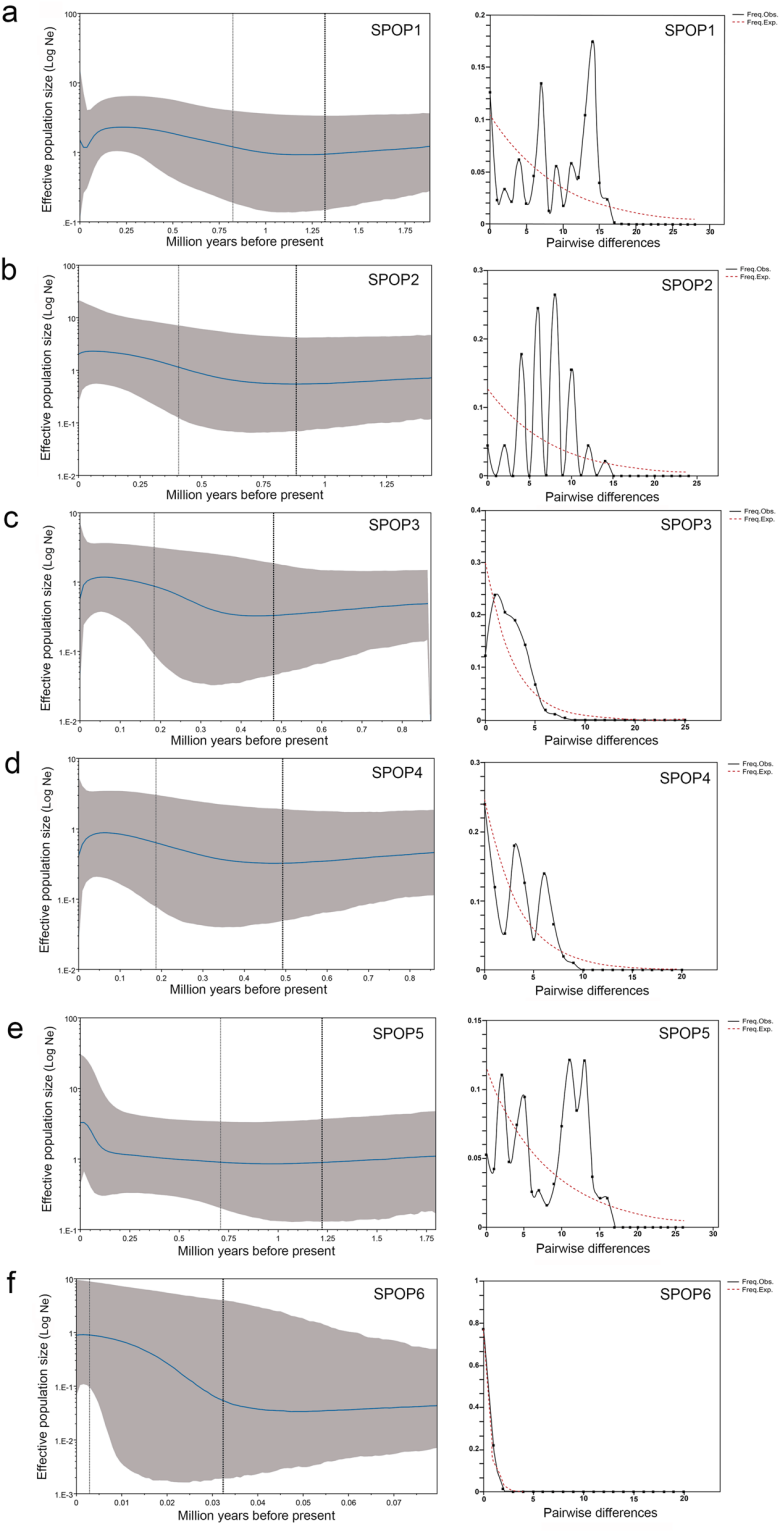
Mismatch distribution (right) and Bayesian skyline plot (BSP) (left) for the six population subdivisions of *H. malabaricus* (BIN BOLD:ABZ3047): (**a**) Lower Amazonas River confluences/Xingu River/Rupuruni River (SPOP1), (**b**) Madeira/Purus Rivers (SPOP2), (**c**) São Francisco/East Atlantic/Eastern Northeast Atlantic/Parnaíba/Itapecuru River basins (SPOP3), (**d**) Guiana Shields drainages (SPOP4), (**e**) Western-Northeast Atlantic/Tocantins basin (SPOP5), (**f**) Tapajós River Basin (SPOP6). BSPs show changes in the effective population size. The thick solid line represents the median estimate and the margins of the surrounding area represent the largest posterior density ranges of 95%.

In the mismatch distribution analysis among the population groups, the SPOP3 and SPOP6 presented a unimodal curve and were suggestive of demographic fluctuations, whereas the SPOP1, SPOP2, SPOP4 and SPOP5 populations displayed multimodal curves (Fig. 5a–f). Bayesian skyline plots (BSP) provided a signal of long-term demographic stability (Fig. 5a–f). For the SPOP1, the BSP showed continuous and discrete historical population size growth approximately 950 thousand years before present (kybp), with a decrease with the onset of the 150 kybp, after which it remained stable for a short time before increasing to the current population size (Fig. 5a). For SPOP2, the BSP plot suggests expansion time estimated onset 600 kybp, followed by a period of stability starting at 200 kybp and shortly after very slight tendency of decrease approximately 50 kybp (Fig. 5b). The SPOP3 and SPOP4 started a period of expansion at 350 kybp, and evidence of a substantial decline started at 50 kybp (Fig. 5c,d). SPOP5 demonstrated a fluctuation, with rapid population expansion starting at 150 kybp (Fig. 5e). For SPOP6, a remarkably progressive increase in population size started at 35 kybp, and the lightest tendency of increase occurred at the start of the Holocene (Fig. 5f).

**Figure 6:**
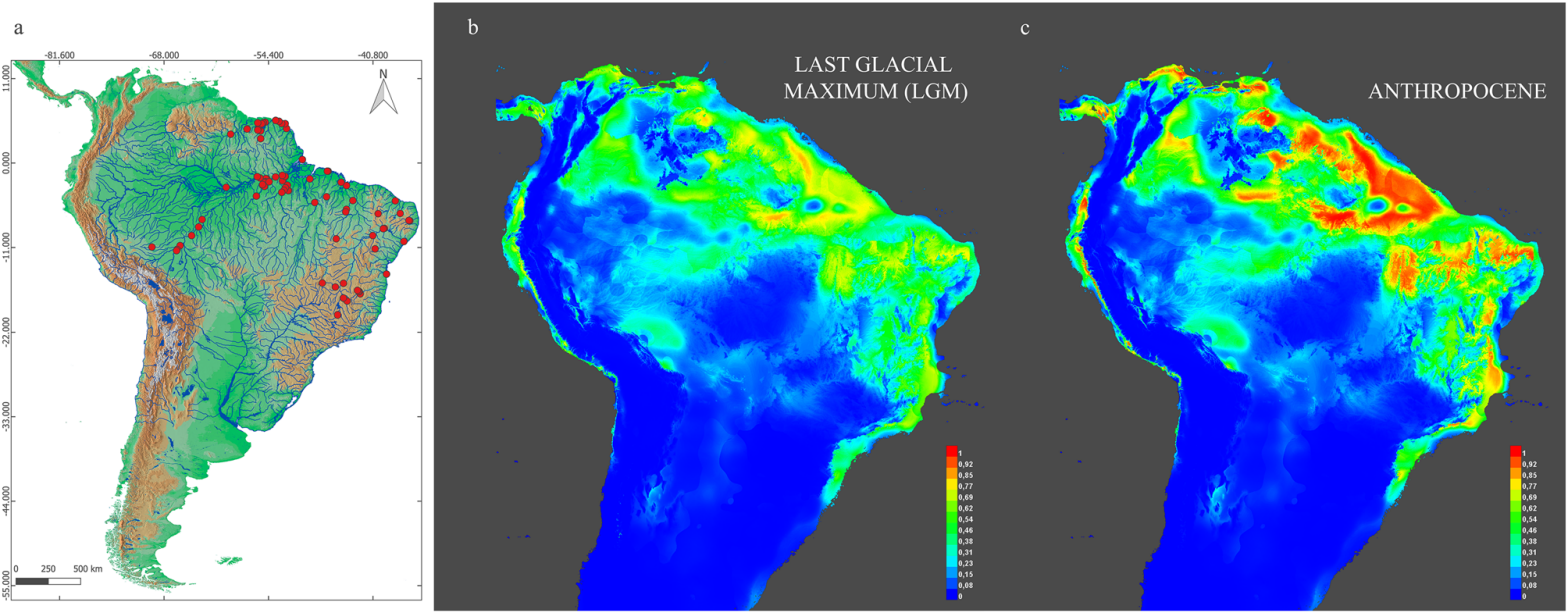
Present distribution of *H. malabaricus* (BIN ABZ3047) (**a**). Bioclimatic model of the most suitable geographic areas for the occurrence of this species in the Last Glacial Maximum (**b**) and Anthropocene (**c**). The warm colors (red, yellow) indicate high probability. The map was done with QGIS v.3.16.8-Hannover (Quantum GIS Development Team, http://www.qgis.org).

For the niche model, the mean diurnal range [mean of monthly (max temp − min temp)] was the most important variable driving the *Hoplias malabaricus* sensu stricto distribution. The other most important variables were the annual temperature range, mean temperature of the wettest quarter, mean temperature of the driest quarter and precipitation seasonality (coefficient of variation). The prediction is *H. malabaricus* was potentially distributed congruent with the present-day geographical distribution. The areas with the highest levels of suitability were eastern Amazon Basin, Guiana Shield and northeast Brazil. Spatial displacements did not seem to occur, but the range size population increased over time (see Fig. 6).

## Discussion

The species of *Hoplias* tend to be morphologically conserved and its taxonomy is still under debate^15,17^. Despite its conspicuous karyotypic variation, cytogenetic markers have shown poor resolution for taxonomy and phylogenetic reconstruction^2,8,9^. Recent progress has been achieved with DNA barcoding coupled to morphological traits (integrative taxonomy), which starts to delineate a clearer picture of the evolutionary history of this group^15,17,52^.

This study revealed that *Hoplias* encompasses at least 23 species and hides new cryptic species in both the *H. lacerdae* and *H. malabaricus* groups. Additionally, taxonomic confusion possibly caused by misidentifications could be detected. For instance, *H. intermedius* (BIN AAB1734) from the Jequitinhonha River (Minas Gerais, Brazil) was deposited as *H. brasiliensis*. Two independent evolutionary units were recovered for *H. aimara* and three for *H. curupira* as previously demonstrated^1^; herein we show that *H. aimara* belongs to the *H. lacerdae* group. Instead of *H. curupira*, which is the most basal clade and could receive a species-group status for the current *Hoplias* classification.

The conservative interspecific genetic distance of 2% for COI sequences was considered imprecise to delimit species from the megadiverse Neotropical ichthyofauna (see Queiroz and colleagues^53^). Using a single marker species delimiting approach we observed deep genetic divergence in *Hoplias*. The species in the *H. malabaricus* group diverged by a maximum of 10.3%, which was enough to discriminate between sister species formally described and lineages from the *H. malabaricus* complex, which was assumed to be independent evolutionary units (ESUs). This magnitude of genetic divergence supports cryptic speciation in the *H. malabaricus* complex, as revealed by multiple species delimitation analyses (BIN, ABGD and GMYC). Therefore, our results corroborate the existence of the *H. malabaricus* complex of cryptic species inhabiting the Amazon Basin and adjacent drainages^2,5,7,8^. Some of these lineages were presented in previous studies^1,9,52^; however, we could recognize six new putative species from the *H. malabaricus* complex (BINs: AAY4779, AAD3630, AEA5279, ADL3159, AEF4663 and AEA4944).

COI deep divergences, higher than 2%, are a marked signature of lineage/species differentiation in *Hoplias*^1,8,9^. Such a phenomenon has been observed between populations from the same and distinct hydrographical basins^8^ and between populations that share identical karyomorphs^9^. *H. misionera* and *H. argentinensis* diverged from their nearest neighbors by 5.6 and 9.0%^15,17^. Jacobina and colleagues^8^ similarly recorded deep divergence (7 to 7.3%) delimiting putative species from *H. malabaricus* lineages in distinct Brazilian hydrographic basins. On the other hand, Cardoso and colleagues^1^ demonstrated that speciation in *Hoplias* may be accompanied by a large range of COI genetic distances, between approximately 1% and 20%. Indeed, *H. mbigua* diverged from its nearest neighbor by only 1.13%^1^.

The BINs AAD3630 and AEA5279 are from Colombia and are composed of individuals from Orinoco basin drainages. The BINs AEA4944 and AEF4663 from the Ventuari-Orinoco were delimited as a single species with ABGD and GMYC. Herein, we consider the taxonomic status of these lineages most imprecise, and their delimitations as putative species must be regarded cautiously. Putative species delimited from a small number of sequences (< 5 individuals) are highly susceptible to bias^31,54^. The ADL3159 lineage was restricted to the Crepori River, a tributary of the middle Tapajós drainage, distantly more than 500 km from the confluence zone between the Tapajós and Amazonas Rivers. This group was supported by distinct methods, and we believe it is a new undescribed species within the *H. malabaricus* complex. Recently, an integrative taxonomic analysis by our team formally describes this taxon (article accepted).

Because of the taxonomic uncertainty of the genus *Hoplias*, particularly in the *H. malabaricus* complex, few studies have focused on population genetics and micro evolutionary processes, which may be caused by imprecise species discrimination under field conditions due to the high genetic diversity (karyomorphs, DNA), morphological similarity and poor taxonomic knowledge. Herein, we successfully identified *H. malabaricus* from cryptic congeners based on DNA barcoding sequences. Although the COI gene has been routinely used for species delimitation^13,55^, it can be useful to explore population genetic structuring in freshwater fishes^12,56^.

From the *H. malabaricus* complex, we detached a clade supported with 65% PP and delimited to BIN ABZ3047, which was interpreted as a single evolutionary unit and tested for genetic structure and taxonomic status validation. This putative species was assumed to be *H. malabaricus* stricto sensu and believed to be representative of *H. malabaricus* (Bloch, 1794). We found a clear genetic structure pattern, initially evidenced by the Bayesian topology that showed multiple subclades, and spatially divergent subpopulations shown with Geneland analyses. High Fst differentiation values and almost 50% of the genetic variation among populations support the genetic structure hypothesis. For comparison, the Fst values of the Tapajos River Basin population (SPOP6), the most differentiated, ranged from 40 to 54%. Aguirre and colleagues^57^ measured a maximum F_ST_ divergence of 20% between *H. microlepis* populations from rivers and artificial impoundments in Ecuador. Due to its wide distribution range and sedentary habits, *Hoplias* species tend to exhibit high population genetic differentiation^9,58^.

Genetic structuring in nonmigratory fish from the Amazon basin dispersed to adjacent drainages has been previously observed in Arapaimidae species, *Osteoglossum bicirrhosum*^59^ and *Arapaima gigas*^60^. We detected a genetic link between populations from Orinoco drainage and the Amazon basin, in the Trombetas River (northern Amazonas drainage). Although, Amazonas and Orinoco systems remain currently isolated, past contacts between them are well documented and such gene flow exchange has been observed in other fishes (*e.g.,* needle fish genus *Potamorrhaphis*)^61^. Therefore, such connections between *H. malabaricus* populations may be explained by lineage sorting taking to the maintenance of ancestral haplotypes.

The SPOP 3 and 6 presented demographic instability and neutrality deviation that was interpreted as a signature of recent expansion. The biological drives in such populations may be adjusted with Pleistocenic phenomena, which span the most recent period of repeated glaciations. The SPOP 3 flows through portions of morphoclimatic domains of the Cerrado, Caatinga and part of the Atlantic Forest, where there is an extensive field of sand dunes in the São Francisco River basin which represents a testimony drier climate in this region in the past. The São Francisco River changed to its present course during the Mindel glaciation (approximately 450 kybp). Presently, it flows towards the north, curving towards the southeast and to the Atlantic Ocean, but this river previously flowed in a different direction, connecting with the Parnaíba River to the Atlantic Ocean^62^, which may explain why this subpopulation was also be formed by samples from the Parnaíba River and the Western Northeast Atlantic basin. Similarly, the highlands in Guiana shields and Andes Mountains accumulated thick ice caps during the Pleistocene glaciations and the Tapajós region experienced long periods of erosion^63,64^. The SPOP6, formed by samples from the lower Tapajós River basin, is consistent within the limits of the Pleistocene (2.5 mybp–11.7 kybp), as shown by with mismatch distribution and population expansion analyses.

Modern Amazonian biodiversity dimensions were achieved during the Neogene (23–2.6 mybp), but the most ancient lineages are probably present since the Paleogene (66–23 mybp) and Late Cretaceous (146–66 mybp)^65–67^. During the middle to late Cenozoic, the Western Amazon basin was a lacustrine habitat, while the eastern and central portions were repeatedly invaded by marine incursions, resulting in the isolation of Guiana and Brazilian Shield tributaries^68^. The river capture dynamics during the Neogene have been proposed as the main force driving the last diversification of aquatic and terrestrial Amazonian taxa that are ecologically restricted to water bodies and riparian forests^65,69^.

The proportion in which animals and plants retain ancestral ecological traits during time scales, that is, niche conservation, is still a controversial theme^70^. The finding of niche conservation is based on many variable aspects of the fundamental niche, taking into account that the rate of adaptation to conditions outside the niche is slower than the rate of extinction. The observation of conservation or niche change is an area of great relevance in the study of niche biogeography and in historical biogeography and in the exploration of patterns and mechanisms of species richness in wide spatial areas^71^. Similar to the study by McNyset^72^, which shows ecological niche conservation for six freshwater fish from North America under a moderate time scale, the present study points out that from the last glacial maximum (LGM) to the Anthropocene the niche remains very close in this taxonomic group. The *H. malabaricus* populations showed demographic evidence of expansion-decline cycles during the Pleistocene, immediately before the LGM. We hypothesize that such demographic fluctuations in *H. malabaricus* may be synchronized with the recent glaciation and the geologic events linked to the formation of the modern Amazonas River system^65,73^.

Currently, *H. malabaricus* sensu stricto is dispersed through the Guiana drainages, Amazon basin, Western Atlantic Northeast basin and São Francisco River basin^1^. These systems experienced connection-isolation cycles during the Pleistocene period driven by climatic fluctuations and geomorphologic forces^1,18,74^. Our paleogeographic model revealed that the hypothetical distribution in the Pleistocene was mostly congruent with the present-day distribution and was influenced by climate change. The geographical distribution of the complex was restricted in the North and Northeast at South America during the LGM, and in the Anthropocene there was a reduction in potential areas. The drainages from the eastern portion of the Amazon basin and coastal drainages in the western Northeast Atlantic basin seem to be important routes for population dispersal.

Geomorphologic forces, mainly plate tectonics, marine incursions/regressions and climate fluctuations, recurrently shaped the Amazon landscapes, driving important processes in aquatic systems and leading to intensive changes in the river courses and river captures. These combined phenomena may be involved in the taxonomic radiation and geographic dispersion of the fish populations throughout the Amazon basin^65–67,75–78^.

To conclude, the data herein indicate that *Hoplias malabaricus* sensu stricto presents a remarkable population structure, which seems to have been caused by past events. The cryptic diversity observed among the *Hoplias malabaricus* species group has conservation and taxonomic implications. Although morphological data have not been evaluated here, our molecular data are necessary to determine whether the mitochondrial lineages can represent distinct species. If to ABZ3047 subpopulations are not shown in the future to be different species, at least the SPOP 3, SPOP 4 and SPOP 6 can be considered as different evolutionarily significant units for conservation purposes. Additionally, even whether *Hoplias malabaricus* sensu stricto constitutes a well-distributed metapopulation in northern South America, its distribution would be much smaller than what is currently attributed to the species; thus, the taxonomy and geographic distribution of *H. malabaricus* should be reevaluated.

## Supplementary Information

Supplementary Information 1.

Supplementary Information 2.

Supplementary Information 3.

## Data Availability

All data generated or analysed during this study are included in this published article and its Supplementary Information files.
